# The Impact of COVID-19 Lockdown Restrictions on Exercise Behavior Among People With Multiple Sclerosis Enrolled in an Exercise Trial: Qualitative Interview Study

**DOI:** 10.2196/42157

**Published:** 2022-11-22

**Authors:** Louise C Palmer, Whitney N Neal, Robert W Motl, Deborah Backus

**Affiliations:** 1 Crawford Research Institute, Shepherd Center Atlanta, GA United States; 2 Department of Health Behavior, University of Alabama at Birmingham Birmingham, AL United States; 3 Department of Kinesiology and Nutrition, University of Illinois Chicago Chicago, IL United States

**Keywords:** multiple sclerosis, exercise, physical activity, COVID-19, COVID-19 lockdown restrictions, telerehabilitation, interview study

## Abstract

**Background:**

During spring and summer 2020, US states implemented COVID-19 pandemic restrictions, resulting in the closure of rehabilitation facilities and, with them, some of the clinical trials that were taking place. One such trial was the Supervised Versus Telerehabilitation Exercise Program for Multiple Sclerosis (“STEP for MS”) comparative effectiveness multiple sclerosis (MS) exercise trial. Although 1 study arm was implemented via telerehabilitation, the comparative arm took place in rehabilitation facilities nationwide and was subsequently closed during this time frame. The experience of the STEP for MS participants provides insights into the impact of lockdown restrictions on exercise behavior by mode of exercise delivery (telerehabilitation vs conventional facility based).

**Objective:**

This study sought to understand the impact of COVID-19 lockdown restrictions on exercise behavior among people with MS enrolled in an exercise trial at the time of the restrictions.

**Methods:**

Semistructured phone and video interviews were conducted with a convenience sample of 8 participants representing both arms of the exercise trial. We applied reflexive thematic analysis to identify, analyze, and interpret common themes in the data.

**Results:**

We identified 7 main themes and 2 different narratives describing the exercise experiences during lockdown restrictions. Although the telerehabilitation participants continued exercising without interruption, facility-based participants experienced a range of barriers that impeded their ability to exercise. In particular, the loss of perceived social support gained from exercising in a facility with exercise coaches and other people with MS eroded both the accountability and motivation to exercise. Aerobic exercises via walking were the most impacted, with participants pointing to the need for at-home treadmills.

**Conclusions:**

The unprecedented disruption of COVID-19 lockdown restrictions in spring and summer 2020 impacted the ability of facility-based STEP for MS exercise trial participants to exercise in adherence to the intervention protocol. By contrast, the participants in the telerehabilitation-delivered exercise arm continued exercising without interruption and reported positive impacts of the intervention during this time. Telerehabilitation exercise programs may hold promise for overcoming barriers to exercise for people with MS during COVID-19 lockdown restrictions, and potentially other lockdown scenarios, if the participation in telerehabilitation has already been established.

## Introduction

### Background

COVID-19 was declared a global health emergency in January 2020 and a pandemic in March 2020 [1]. Governments worldwide, including in the United States, subsequently implemented public health emergency measures, including legally enforced closures and limitations on the maximum capacity of places where people congregate (ie, schools, stores, and recreational facilities), physical distancing, mask wearing, limitations on nonessential domestic and international travel, and self-isolation or quarantine requirements [2]. These measures disrupted participation in work, education, travel, recreation, exercise, and physical activity (PA), with potentially significant physical and mental health implications, especially for people with disabilities, including multiple sclerosis (MS). Indeed, 2 years after the lockdown restrictions were lifted, research indicates that vulnerable populations, including people with MS, experienced heightened social isolation, increased depression and anxiety [3,4], and a lack of access to health services [5-7].

MS is a chronic neurological disease of the central nervous system with prevalence among nearly 1 million adults in the United States and 2.5 million adults worldwide [8]. Damage within the central nervous system yields the myriad of symptoms experienced by people with MS, resulting in functional limitations, cognitive dysfunction, and reduced quality of life (QOL) [9]. PA and exercise can improve walking, balance, fatigue, depression, and QOL for people with MS [10]. However, people with MS are more inactive than the general population [11] and experience many barriers to exercise, including a lack of MS-adapted exercise protocols [12].

### Objective

Both facility-based and telerehabilitation (telerehab) exercise training have yielded positive results in people with MS. However, the facility-based and telerehab modes of delivering exercise have not been compared head to head. Comparing the outcomes of delivering the same exercise intervention in a facility (facility based) and in the home or community using a telerehab approach would be an important step toward informing people with MS about their exercise options. The Supervised Versus Telerehabilitation Exercise Program for Multiple Sclerosis (STEP for MS) comparative effectiveness trial was designed to address this knowledge gap by assessing the effectiveness of the *Guidelines for Exercise in People With Multiple Sclerosis (GEMS)* exercise protocol [13,14] for people with MS delivered via telerehab compared with in a conventional facility setting. The primary research question of the STEP for MS study was as follows: “In people with MS, does an evidence-based individualized exercise program delivered via telerehab yield comparable benefits for improving walking, mobility, participation, and QOL when compared with delivery via a conventional, facility-based approach?” By the beginning of March 2020, the STEP for MS trial had enrolled 217 participants across 8 study sites throughout the United States.

In the United States, governors implemented pandemic restrictions on a state-by-state basis; however, by the end of March 2020, all STEP for MS study sites were temporarily closed to in-person research activities. Studies indicate that the full or partial closure of fitness and rehabilitation facilities and parks and the continuation of self-isolation directives for medically vulnerable persons through early summer 2020 decreased the PA levels among people with MS [15,16]. Indeed, 1 study estimated that almost half of the people with MS decreased their PAs during the COVID-19 pandemic [15]. Studies assessing the impact of COVID-19 restrictions on PA and exercise among people with MS use a cross-sectional research design and survey methodology, which do not delve into the unique perceptions and experiences of people with MS [15-18]. A deeper understanding of these issues can inform the development of programs that will more effectively address the unique needs of people with MS. To address this gap, this study used a qualitative research design and explored the impact of COVID-19 lockdown restrictions on exercise behavior among a small convenience sample of adults with MS enrolled in STEP for MS. The study further explored whether participants’ experiences during lockdown restrictions had implications for the design and implementation of future exercise trials. Specifically, our study sought to answer the research question, “What impact, if any, has the COVID-19 pandemic had on the exercise behaviors of people participating in the STEP for MS trial?”

## Methods

### Study Setting and Design

This research was an ancillary qualitative study conducted as part of the larger STEP for MS multisite trial (NCT03468868). The STEP for MS trial compares the effectiveness of a 16-week evidence-based, individualized exercise training program (GEMS) delivered via telerehab with that of the same program delivered in a facility among people with MS who have walking dysfunction and mobility disability (assumed to be due to their MS) [19]. The STEP for MS trial began recruitment in September 2018 and followed a 2-stage, randomized choice design for examining improvements in walking performance in people with MS. Specifically, the participants were first randomized into either the assigned or choice arm. The second stage of randomization was then applied only to those in the assigned group, whereby participants were randomly assigned to either the facility-based or telerehab intervention arm. Within the choice group, the participants made a preferred selection of facility (Guidelines for Exercise in People With Multiple Sclerosis-Supervised [GEMS-S]) or telerehab (Guidelines for Exercise in People With Multiple Sclerosis-Telerehabilitation [GEMS-T]).

Both intervention arms received an individualized exercise prescription that consisted of aerobic exercise focused on walking as the modality and strength training exercises targeting the lower body, upper body, and core muscle groups. The participants in the GEMS-S group completed exercise sessions in person under the supervision of an MS exercise behavioral coach based at 1 of the 8 study sites. By contrast, GEMS-T is delivered remotely via Zoom by exercise behavioral coaches located at the University of Alabama at Birmingham (UAB) Intervention Center. Specifically, the GEMS-T coach oriented the participants to the exercise prescription but did not provide remote supervision during the aerobic and strength exercise training sessions. Instead, the GEMS-T participants had the option of following written or video instructions for the exercises and completing them in their home, at a gym, or on walking trails in their community. In addition, the participants in both groups received one-to-one sessions with behavioral coaches at regular intervals. These sessions focused on the guidance on and oversight of appropriate exercise techniques, discussion of action planning and self-monitoring, and delivery and discussion of newsletters designed to optimize exercise adherence. To ensure the fidelity of intervention delivery across sites, the STEP for MS trial used training and quality checks, including initial and ongoing site training on the exercise training program and social cognitive theory (SCT) principles of behavior change for exercise, weekly meetings with the intervention center project coordinator and all collaborating site behavioral coaches, and audits of one-to-one behavioral sessions across sites. Further details of the intervention protocol and fidelity measurements have been previously reported [19].

Delivery of the STEP for MS intervention began in October 2018; however, the COVID-19 pandemic meant that many of the intended intervention procedures described earlier were no longer feasible for GEMS-S participants as of March 18, 2020, when restrictions were put into place. Specifically, the closure of study sites for clinical trials and studies not related to COVID-19 prevented sites from conducting the GEMS-S in-person exercise training visits. Thus, the STEP for MS trial team worked rapidly to implement adaptations to intervention delivery for the participants in the GEMS-S condition. After an initial 3-week pause in intervention activities for the GEMS-S participants, activities resumed via internet-based and phone-based supervised exercise sessions using web conferencing software (eg, Zoom [Zoom Video Communications, Inc]) instead of face-to-face sessions from early April 2020.

This ancillary qualitative study recruited participants from 2 of the 8 study sites (UAB and Shepherd Center), where lockdown restrictions were implemented for a brief period spanning April 2020, followed by an advisory order only in Alabama through May 2020, but a mandatory stay-at-home order was implemented for people at increased risk in Georgia through May 2020 [20].

### Philosophical Assumptions

This research was underpinned by ontological relativism and epistemological constructivism [21,22]. Ontological relativism asserts that reality is a subjective experience, whereas constructivist epistemology is underpinned by the belief that knowledge is constructed through personal interactions with the social and physical environment and that the researcher has an active role in the construction of knowledge generation [21,22]. These philosophical underpinnings informed an interpretivist paradigm, whereby we recognized that the purpose of the research involved identifying various subjective and multifaceted perceptions of the impact of the pandemic on each participant’s exercise behavior.

### Recruitment

This qualitative interview study was approved by the Shepherd Center Research Review Committee and conducted as a part of the ongoing STEP for MS comparative effectiveness trial [19]. The qualitative study used purposeful sampling strategies, specifically convenience, criterion-based, and quota-based sampling techniques. The participants of this study were a convenience sample enrolled at either our Shepherd Center or UAB study sites. Criterion-based sampling strategies specifically seek individuals who possess certain characteristics that speak to the research questions. The first and second authors (LCP and WNN) contacted participants who were completing the exercise intervention portion of the STEP for MS trial when COVID-19 restrictions were imposed (March to April 2020) or who had recently concluded the 16 weeks of exercise and who were enrolled at either the Shepherd Center or UAB study location. This sampling method allowed the recruitment of persons with MS who could (1) provide rich data from current personal experiences for addressing the research questions [23] and (2) yield data that were detailed and in-depth enough to inform meaningful and impactful results relevant to the design and implementation of future exercise trials [24]. Quota-based sampling seeks an equal representation of participants [25]. We targeted the recruitment of 5 persons per study location (Shepherd Center and UAB) with equal representation between the study arms (home based [GEMS-T] and facility based [GEMS-S]) for a broad cross-section of feedback on the impact of COVID-19 lockdown restrictions on exercise behavior. The first and second authors (LCP and WNN) contacted 14 participants who met the inclusion criteria stated earlier (ie, those who were currently completing or recently completed the 16-week exercise intervention and were enrolled at either the Shepherd Center or UAB study location). Of these 14 people, 9 (64%) expressed interest. One of the participants from UAB was lost to follow-up or did not attend the interview. We believe that our sample of 8 participants, although small, is acceptable within the context of this pragmatic study and provides sufficient “information power” to answer our research question [26]. Information power refers to the amount of information the sample provides that is relevant for answering the research question. The greater the amount of information contained in the sample, the smaller the number of participants required. Although small, we also believe that our participants provided quality information, another metric for assessing the adequacy of sample size in qualitative research [27].

### Data Collection

Data were collected through one-to-one semistructured interviews conducted either on the web (3/8, 38%) or via phone (5/8, 62%) by trained interviewers (LCP and WNN). The interview guide is provided in the Multimedia Appendix 1. We had the camera on for interviews conducted on the web but did not have video for those conducted via phone. There are pros and cons to different interviewing media (Saarijärvi and Bratt [28]), but a mixed format has been used successfully in other published studies (refer to, eg, Neal et al [29]) and likely does not affect the study findings [28].

The coauthors developed the interview guide through engagement with the literature, guidance from an expert in qualitative research methods, and discussion with the third and fourth authors (RWM and DB) to address the overarching research question, “How has the COVID-19 pandemic impacted your exercise?” The interview guide adopted a chronological approach to explore the levels of participant exercise before the COVID-19 pandemic, during lockdown restrictions, and at the time of the interview (May 2020, when some but not all restrictions had been lifted). After establishing the general pattern of exercise over this period, the interview guide used a strength- and barrier-based approach to explore what helped and hindered exercise during the lockdown. The interviewer (either LCP or WNN) presented questions and prompts in a semistructured format, allowing the participants the freedom to elaborate when discussing experiences that were important to them but affording the interviewer the opportunity to focus on areas of interest. Questions encompassed a range of topics regarding participants’ experiences and perceptions related to exercise and coping during the COVID-19 pandemic such as, “What has helped or hindered exercise during the pandemic?” and “What additional resources would have been helpful during the pandemic?” Then, the participants were asked, “What concerns do you have about remaining in the STEP for MS trial as the pandemic continues?” and “How has COVID-19 changed your thoughts on participating in future exercise studies?” Questions about continuing in the STEP for MS trial and future exercise trials were asked after other questions pertaining to perceptions related to exercise and coping during the pandemic and were open-ended, thereby allowing the participants the freedom to express their views for or against future participation in exercise trials.

### Ethics Approval

The participants provided verbal consent for taking part in the interview with audio recording in accordance with the Shepherd Center’s institutional review board approval process (protocol number 738; Shepherd Center’s Institutional Review Board credentials: FWA00000642 and IORG0001082), and the participant names were removed from the transcripts and replaced with pseudonyms (Textbox 1).

Raw data worth >4 hours were collected, and interviews lasted between 23 and 43 minutes. Clinical and demographic data were collected as part of the STEP for MS trial baseline data collection.

**Participants’ group assignments and pseudonyms.**

**Guidelines for Exercise in People With Multiple Sclerosis-Telerehabilitation**

- Kimberly (research site A)
- Sarah (research site A)
- Dana (research site B)
- Leslie (research site B)
- Maureen (research site B)

**Guidelines for Exercise in People With Multiple Sclerosis-Supervised**

- Anita (research site A)
- Gayle (research site B)
- Wendy (research site B)

### Data Analysis

To understand the meaning of the data, we applied reflexive thematic analysis (RTA), a flexible interpretative approach to qualitative data analysis for identifying, analyzing, and interpreting common themes in the data [30]. The data were analyzed using predominantly inductive RTA, whereby codes and themes were generated from the participant testimonies. A degree of deductive analysis was used to ensure that the data-based meanings emphasized in open coding contributed to the production of themes that were meaningful to the research questions. To ensure rigor, data analysis was completed by LCP and WNN through six iterative phases: (1) familiarization with data, (2) generating initial codes, (3) searching for themes, (4) reviewing themes, (5) defining themes, and (6) final analysis. LCP and WNN became immersed in the data by conducting interviews, rereading transcripts, taking notes of initial ideas related to the research question, and creating initial codes (steps 1 and 2). Initial themes were generated by creating a list of codes for each participant, sorting and collating these lists into potential themes, and then placing similar codes in the same group (step 3). To ensure trustworthiness, LCP and WNN met regularly to discuss the meaning of the codes and themes in relation to the research questions. Subthemes were generated when necessary to demonstrate the hierarchy of meanings within a theme. Themes were then refined through discussions between the research team members (including RWM and DB) regarding the appropriateness of each theme in relation to the research questions (step 4). During steps 5 and 6, the themes were defined and named in a way that explained the data content and answered the research question, and the results were collated into a written narrative with data extracts to illustrate each theme, which will be presented in the *Results* section.

### Ensuring Rigor

To ensure rigor and trustworthiness throughout the qualitative research process, we adopted a relativist approach, whereby we chose study-specific markers of evaluative quality from Smith and Caddick’s ongoing list [31]. We chose the evaluative markers of substantive contribution, worthy topic, and transparency. First, we demonstrated the worthiness of our topic by justifying in the introduction why examining the experiences and perceptions of coping and exercise during the COVID-19 pandemic among people with MS could advance the understanding in this area and have significant implications for practice. Second, we ensured substantive contribution by identifying a gap in knowledge within the field of MS and exercise, which, if answered well, could meaningfully contribute to our understanding and appreciation of exercise in MS. Finally, we sought to be transparent by completing an audit trail, whereby the first and second authors served as “critical friends” throughout the analytical process.

## Results

### Overview

The mean age of the interview participants was 51.5 (SD 6.5; range 45-60) years; 50% (4/8) of the participants identified as Black and 38% (3/8) as White; and all were female (8/8, 100%). Participants represented a range of MS types and disability status, as reflected in the Patient-Determined Disease Steps and the Expanded Disability Status Scale presented in Table 1. Both groups had similar characteristics in terms of these disease measures. A total of 5 participants were enrolled at the Shepherd Center site, and 3 were enrolled at UAB. Six participants were in the process of completing the exercise intervention at the time of lockdown restrictions, and 2 participants (1 per site) had recently concluded the intervention.

The experiences and perceptions related to exercise during the COVID-19 lockdown restrictions were extensive, complex, and contrasting among the participants. All the participants discussed barriers to exercise during the pandemic, and perceptions differed depending on whether they were in the GEMS-S or GEMS-T study arm. The following sections outline 2 narratives based on these study arms and propose potential reasons for the existence of 2 different narrative paths. Briefly, the GEMS-S participants experienced disruption to exercise during the lockdown restrictions, whereas the GEMS-T participants adapted to the new exercise conditions and continued to exercise seamlessly. Through RTA, LCP and WNN identified a total of seven themes related to the exercise experiences of people with MS during the COVID-19 pandemic (Textbox 2): (1) GEMS-T—disruptions and adaptations to the exercise environment, (2) GEMS-T—applying GEMS strategies to adhere to exercise, (3) GEMS-T—exercise as a coping mechanism, (4) GEMS-S—environmental barriers to exercise, (5) GEMS-S—loss of social support reduced the self-motivation to exercise, (6) GEMS-S and GEMS-T—request for resources to support home-based exercise, and (7) GEMS-S and GEMS-T—COVID-19 pandemic**–**related concerns.

**Table 1:** Demographic and clinical characteristics.

Characteristics			GEMS-S^a^ (n=3)		GEMS-T^b^ (n=5)
Sex (female), n (%)			3 (100)		5 (100)
**Age (years)**					
	40-49, n (%)	1 (33)		3 (60)	
	50-59, n (%)	2 (67)		1 (20)	
	60-69, n (%)	0 (0)		1 (20)	
	Value, mean (SD)	52.7 (6.5)		50.8 (7.2)	
**Race, n (%)**					
	African American or Black	3 (100)		1 (20)	
	White	0 (0)		3 (60)	
	Chose not to answer	0 (0)		1 (20)	
Ethnicity (non-Latino), n (%)			3 (100)		5 (100)
**MS^c^ type, n (%)**					
	Relapse-remitting MS	3 (100)		3 (60)	
	Secondary progressive MS	0 (0)		2 (40)	
**PDDS^d^**					
	3—Gait disability, n (%)	1 (33)		0 (0)	
	4—Early cane, n (%)	2 (67)		1 (20)	
	5—Late cane, n (%)	0 (0)		2 (40)	
	6—Bilateral support, n (%)	0 (0)		2 (40)	
	Value, median (IQR; range)	4 (0.5; 3-4)		5 (0.25; 4-6)	
**EDSS^e^**					
	4.5—Relatively severe disability, n (%)	0 (0)		1 (20)	
	5—Disability affects daily routine, n (%)	1 (33)		0 (0)	
	6—Assistance required to walk, n (%)	2 (67)		4 (80)	
	Value, median (IQR; range)	6 (0.5; 5-6)		6 (0; 4.5-6)	
**Randomization status, n (%)**					
	Choice	1 (33)		3 (60)	
	Assigned	2 (67)		2 (40)	

^a^GEMS-S: Guidelines for Exercise in People With Multiple Sclerosis-Supervised.

^b^GEMS-S: Guidelines for Exercise in People With Multiple Sclerosis-Telerehabilitation.

^c^MS: multiple sclerosis.

^d^PDDS: Patient-Determined Disease Step.

^e^EDSS: Expanded Disability Status Scale.

**Themes and subthemes along with their definitions.**

**Theme 1: Guidelines for Exercise in People With Multiple Sclerosis-Telerehabilitation (GEMS-T)—disruptions and adaptations to the exercise environment**

- The GEMS-T participants made changes to their environment to facilitate continued exercise during lockdown restrictions.

**Theme 2: GEMS-T—applying Guidelines for Exercise in People With Multiple Sclerosis (GEMS) strategies to adhere to exercise**

- The GEMS-T participants’ use of strategies learned through the exercise intervention facilitated continued exercise during lockdown restrictions, and accountability was gained from participating in the trial.

**Theme 3: GEMS-T—exercise as a coping mechanism**

- The exercise study provided the GEMS-T participants with a way to cope with boredom and anxiety during the lockdown restrictions.

**Theme 4: Guidelines for Exercise in People With Multiple Sclerosis-Supervised (GEMS-S)—environmental barriers to exercise**

- The GEMS-S participants identified barriers to exercise in their physical environments.

**Theme 5: GEMS-S—loss of social support reduced the self-motivation to exercise**

- The GEMS-S participants described how the lack of in-person coaching and peer support during the lockdown restrictions decreased their motivation to exercise. Social support is a latent theme, which facilitates self-motivation, a semantic theme. The GEMS-S participants recommended an increase in social support when transitioning from exercising in the facility to exercising at home.

**Theme 6: GEMS-S and GEMS-T—request for resources to support home-based exercise**

- Both GEMS-S and GEMS-T participants requested more resources to support home-based exercise.

**Theme 7: GEMS-S and GEMS-T—COVID-19 pandemic–related concerns**

- These are factors directly related to the COVID-19 pandemic in general that prevented or hindered the participants in both groups from staying physically active.

**Theme 7.1: perceived vulnerability determines the level of caution when exercising**

- This subtheme reflects participants’ general fear about keeping healthy and safe during the pandemic and fears of catching COVID-19 while exercising in public spaces.

**Theme 7.2: COVID-19 restrictions deter exercise**

- This subtheme reflects participants’ discomfort with exercising indoors and dislike of mask wearing while exercising inside and outside.

### Theme 1: GEMS-T—Disruptions and Adaptations to the Exercise Environment

Several barriers to the physical environment, such as lack of home aerobic exercise equipment and hilly neighborhood terrain, impacted exercise participation; however, the GEMS-T participants explained how they adapted to the new limitations imposed by the lockdown restrictions and continued their aerobic exercise. For example, Sarah, concerned about catching COVID-19 in outdoor public spaces, changed her walking location from a park to her neighborhood. After learning about the apartment complex’s first positive COVID-19 case, she further restricted her exercise to spaces directly around her apartment building.

Similarly, as the garden where Dana previously walked was closed during the lockdown restrictions, she pivoted to using her home exercise gym. Before the lockdown restrictions, Leslie, a teacher, walked on the school exercise track after classes, and, subsequently, she switched to walking on trails by her house:

[There is] a park a block away. And we’re in kind of an isolated area, so it’s super safe and easy for us to go out for a walk. We actually have trails around our house.

Leslie

Finally, Maureen attempted to walk around her neighborhood but found it more exhausting than walking on the treadmill in her subdivision’s indoor gym and, therefore, continued using the gym, which she described as clean and empty:

They have two treadmills, a bike and a stair master, that’s it...I’m the only one using it. And then, they left that container of wipes and things to wipe down the equipment that I need...So far, I’ve never, in all the months since March, I’ve never seen another person in there when I was in there.

Maureen

Overall, 80% (4/5) of GEMS-T participants continued exercising during the lockdown restrictions without a break. When classes moved to the web, Leslie found the first week of teaching remotely challenging, with no time for exercise. However, she quickly adapted and resumed exercising:

After that week, the pandemic didn’t make it any harder for me, the pandemic itself. It’s just that week was so overwhelming.

Leslie

### Theme 2: GEMS-T—Applying GEMS Strategies to Adhere to Exercise

The GEMS-T participants described using a variety of tools and strategies learned through the STEP for MS trial to adhere to exercise during the pandemic-imposed lockdown restrictions. For example, when Sarah changed her exercise location from a park to what she described as a less inspiring location (her apartment complex), she listened to music for motivation, a coaching suggestion:

It’ll make it seem as if the time is not dragging.

Sarah

The STEP for MS self-monitoring tools provided both motivation and accountability to the GEMS-T participants during the pandemic. For example, Maureen called the progress log her “accountability partner” and explained how it enabled her to see progress in her step count over the course of the study:

It [the progress log] kind of lets me know when I started. And I mean, when I started, I was lucky to do 500 steps. Now I’m doing 1700 steps...So it’s nice to see the improvement.

Maureen

Likewise, Dana stated the following:

It was good for me to keep track of what I was doing and to have the goal of always meeting the next step up.

Dana

Several participants noticed an improvement in MS symptoms and weight loss as a result of participating in the exercise program. These benefits of exercise became self-rewarding and increased the participants’ motivation to continue exercising. Dana said the following:

It made me more committed...because I can see improvement.

Dana

Similarly, Maureen lost 20 pounds and noted the following:

My endurance is better; I can go all day.

Maureen

The GEMS-T participants considered the commitment they made to the study as key to continued adherence to the exercise program throughout the lockdown:

I think the accountability of participating in a program and keeping you connected with what you should be doing and what’s new, is actually a positive aspect of participating in the trial...It’s just a commitment. I made a promise and I’m doing what I said I would do.

Dana

It (the trial) kept me accountable...I was like, my data won’t be accurate...you’d better do it [exercise].”

Leslie

### Theme 3: GEMS-T—Exercise as a Coping Mechanism

The participants from the GEMS-T group described how participating in the exercise trial provided a positive distraction during the lockdown restrictions and helped them cope with pandemic-related anxiety and depression. For example, Dana gave the following explanation:

I was just very thankful that I had this program to do at home, and also it made me thankful that I had something to concentrate on as well...It gave me something.

Dana

Similarly, Maureen noted how she exercised beyond the intervention requirement of 2 times a week to pass the time:

I’ve actually been doing it [exercise] four or five times a week...because there’s nothing else to do.

Maureen

Dana also specifically attributed exercise to helping her cope with the anxiety associated with the pandemic:

So, the anxiety [of the pandemic] is there and yes, I think maybe exercise helps me clear my head...while I’m on the treadmill, I can think through things cohesively.

Dana

### Theme 4: GEMS-S—Environmental Barriers to Exercise

In contrast to the GEMS-T participants, those in the GEMS-S group described multiple barriers to exercise associated with their physical environment, as they transitioned from exercising in the facility under the supervision of a coach to home-based exercise. Although the GEMS-T participants described making adaptations to their physical environment to ensure continued engagement in exercise, several GEMS-S participants considered their outside terrain to be unconducive for aerobic exercise, making walking difficult. Indeed, 2 GEMS-S participants had mostly stopped exercising at the time of the interviews. Anita explained that she had planned to use her gym membership to continue exercising after the study ended and that sometimes she walked inside Walmart for exercise, but neither option was available during the lockdown restrictions:

But you know, of course since this happened, nobody wants to go into a gym or whatever, no, I don’t feel comfortable.

Anita

Meanwhile, walking outside was challenging because of the hills in Anita’s neighborhood:

The neighborhood I live in is so hilly...If the area was more flat, I would be more apt to just go outside and walk, you know?

Anita

For Gayle, the transition from walking on a treadmill at the facility to walking outside in her neighborhood was challenging:

I didn’t realize how different it would be walking outside versus walking on the treadmill...It’s [the outside terrain] not flat like the treadmill, which is something I didn’t really contemplate, or think about, or make allowances for. And I was still trying to walk the same pace. But I got kind of like upset with myself like, “You’re not walking as fast.”

Gayle

However, later in the interview, Gayle alluded to a confluence of factors that combined to discourage her from walking outside:

If I had a treadmill, I would be able to do that. It’s just having to go outside. We put on our masks, and we get going. And then, as soon as I get out of my house, we have to go around a curve, and then it goes up a hill. And then the other part of my house is in a cul-de-sac, so I don’t have any option except to go up this little hill. And then, when you’re going up the hill, and you start breathing faster, and then you feel like the mask is, you know, you gotta take it out, take your face out of the mask, because you need to breathe better. It’s just not...it doesn’t feel right.

Gayle

She continued to talk about the need to take her cane with her to walk outside:

It’s more difficult for me to walk up and down the hill because I have to take my cane with me if I’m outside. When I was on the treadmill, I could just walk without even holding on, or if I needed to, I could hold on, but outside I’ve been slower. I have to take my cane.

Gayle

For Gayle, these barriers resulted in her dropping the walking portion of the exercise altogether during the lockdown restrictions. Gayle wished that she had been advised on how different walking outside would be compared with the controlled treadmill experience in the gym:

Just let them know...that it may not be the same walking on a treadmill, it may not be the same as walking outside, but make sure that you know that the amount of steps you take really doesn’t matter. Just walk.

Gayle

Although Wendy had stopped exercising at the time of the interviews because of ill-health, she also expressed reservations about walking in her neighborhood because of the terrain and safety concerns. Instead, Wendy would use the treadmill at her home:

I would walk outside, and I also have a treadmill, but because of the stability to walk outside, I don’t walk outside as much. I actually kind of stopped, and then our neighborhood had rumors that there were coyotes in the neighborhood. And so, because I was mostly walking by myself, I didn’t feel like that was safe. So, I really started to do the treadmill inside more than anything...And my driveway, it’s kind of a hill. I could barely move my way up the drive way. So, I said, this is just not a good idea, so that’s really when I made the final decision, that I’ve got to do this inside.

Wendy

### Theme 5: GEMS-S—Loss of Social Support Reduces the Self-motivation to Exercise

Moving from facility to home during the lockdown restrictions disrupted the in-person social connections during exercise that the GEMS-S participants had become accustomed to. Anita talked at length about lacking the motivation that she had in the facility to exercise at home during the lockdown:

And I really just haven’t been motivated, but I will put on some music and just do it...It’s just in my mind, I’ve just like, I know I need to do this, but I don’t really feel like it.

Anita

Similarly, Gayle gave the following explanation:

It [lockdown restrictions] took all of my momentum away because I haven’t exercised really throughout my life, but I prayed that I would be able to come to the facility, because I knew that I really needed that. I needed that support.

Gayle

For both Anita and Gayle, the loss of in-person coaching and peer support that they received at the facility appeared to be connected to a loss of motivation to exercise at home. For example, Anita gave the following explanation:

Cause I mean, that was somewhere I could go the two days out of the week. Me and him [the coach] would laugh and talk...So, even though I was working, I was exercising, but with his method, how he would just be talking to me and we would be talking.

Anita

Gayle noted the lack of both social support from peers and in-person coaching support as driving her lack of motivation to exercise at home:

At first, I couldn’t even do 10 [minutes of walking], I thought I was going to die. But I got up to 30 and I really felt encouraged. And I would see other people at the facility that maybe had...less level of functioning mobility than I had. And so, I was just encouraged like, “You can do this. Look at them, they’re doing it.” And I really thought I was doing well. And then when I had to do it at home, I just didn’t have that. I mean, Irene [the GEMS-S coach] is great. It wasn’t her at all. I don’t know, I just lost it.

Gayle

Importantly, here, Gayle noted that her GEMS-S coach was supportive in the web-based environment; therefore, it was not the lack of coaching per se that she missed. Rather, it was the overall environment consisting of peers with disabilities exercising together with in-person coaching. Gayle expounded the following:

In the facility, even though we weren’t working out together, it was like we were all on the same team...

Gayle

I love to see the other people at the center, and if their disability [was] maybe a little bit more pronounced than mine, it’s just “look at how she’s doing. She’s doing so good...”

Gayle

To have the coaches supporting you [in the facility], and it just felt like I had a purpose, more so than being by myself...

Gayle

For Gayle, it was not simply receiving peer support that made the difference but also providing it to others:

It was good to hear the encouragement [in facility], or to be able to encourage somebody else.

Gayle

### Theme 6: GEMS-S and GEMS-T—Request for Resources to Support Home-Based Exercise

Unsurprisingly, the GEMS-S participants recommended increased social support and accountability when transitioning from exercising in the facility to exercising at home. Anita recommended “some kind of alarm or some kind of alert or message or something” to hold her accountable to exercise. Other GEMS-S participants enjoyed exercising alongside their peers while in the facility and would have liked a way to remain connected in the web-based environment. For example, Maureen suggested the following:

A visual Zoom program would be more beneficial because you would develop relationships more with people that way I guess.

Maureen

Both GEMS-S and GEMS-T participants requested more resources to support home-based exercise. These resources include exercise demonstration videos and aerobic exercise equipment such as a treadmill. For example, Anita said the following:

I got my equipment [resistance bands and pedometer], you know, the stuff, but of course the equipment [treadmill] we don’t have it here.

Anita

Anita also requested exercise videos:

If I can find something, I’ve got a smart TV. Like if there was some kind of YouTube channel or somebody with some little exercises or something.

Anita

Both Sarah (GEMS-T) and Gayle (GEMS-S) noted the need for a treadmill:

I kind of wish I had a treadmill because I had been thinking about it for a long time. And I‘m just like, I feel like even if I do 10 minutes on the treadmill, that’ll be equivalent to me walking in a neighborhood or something like that.

Sarah

The problem is, I just have to buy a treadmill.

Gayle

### Theme 7: COVID-19 Pandemic–Related Concerns

At the time of the interviews, much was still unknown about how COVID-19 was transmitted and how COVID-19 might impact people with MS, and a vaccine remained far away.

Theme 7 covered factors related to the COVID-19 pandemic that hindered participants in both groups from exercising. This theme was divided into 2 subthemes.

### Theme 7.1: Perceived Vulnerability Determines the Level of Caution When Exercising

As people with a chronic illness who may experience lowered immunity to disease in general, the participants were cautious in their daily activities, including exercise, to prevent contracting COVID-19. When deciding whether to exercise in a public setting (indoor and outdoor), the participants took precautionary measures based on their perceived vulnerability to COVID-19. For example, Anita said the following:

I’m just kind of leery about it, because you don’t know if this is airborne outside.

Anita

Likewise, Sarah was wary of being around other people outside:

And then when I found out that someone in the community had it, I’m just so afraid. By like with my immune system being compromised, like how it is, I catch a cold in a minute or something, so I’m just like, I’m kind of scared. I took a chance. I just really didn’t want to take, so I’m not ready to go quite yet to the park.

Sarah

Gayle expressed how the uncertainty about COVID-19 transmission led to her wearing a mask while exercising outside and how this also made walking more difficult:

And then they [news anchors] were saying that it can be in the air. And then one show said that the wind carries it. And that’s what I was afraid of. We would always go outside with our masks...but then if you’re trying to walk, and you have this mask on, you feel like you’re smothered a little bit.

Gayle

### Theme 7.2: COVID-19 Restrictions Deter Exercise

Participants disliked wearing masks while exercising both indoors and outdoors but felt unsafe exercising inside gyms (which had just begun to reopen) without wearing masks:

I’m not going to go in there [the gym] without a mask on, so I won’t be doing that.

Anita

...with the face mask and the heat, that was strange, but it impacted where I wouldn’t want to go to a park where I would normally go on a walk inside. I wouldn’t want to go.

Sarah

When asked whether Gayle would walk more if the terrain was flat, she responded that the mask was just as much of a barrier to exercising outside:

Yeah. I think if it was something similar to the treadmill, yeah. And if I didn’t have to wear the mask, probably because like when you start walking, and start breathing faster, and I don’t know, it just feels like you can’t really exchange oxygen as well.

Gayle

## Discussion

### Principal Findings

This study contributes to the literature by detailing how participants in 2 different arms of an MS exercise trial adhered to the exercise intervention during the COVID-19 lockdown restrictions and immediately after the restrictions were lifted. The participants randomized to the facility-based arm (GEMS-S) of the STEP for MS trial were more likely to describe barriers to continued exercise such as the lack of social support or home aerobic exercise equipment, whereas telerehab participants (GEMS-T) were more likely to highlight positive experiences, including adaptations to their exercise environment and using exercise as a coping mechanism. Our findings indicate important considerations for researchers and providers about how to meet the exercise needs of people with disabilities in the new pandemic reality and for investigators planning future exercise interventions.

A total of 2 different narratives emerged from our findings. During the lockdown time frame, the GEMS-T participants continued apace with the exercise intervention without interruption. By contrast, the GEMS-S participants described how the shift to remote exercise disrupted their progress and motivation to the point of inertia. Although the participants in both groups continued with resistance training to some extent using the provided resistance bands, the walking component of the intervention proved to be more difficult to maintain for the GEMS-S participants. Perhaps because the GEMS-T participants already had several weeks of at-home exercise established by the start of lockdown restrictions, exercise was routinized enough to accommodate the disruption, even when restrictions necessitated a change in the aerobic exercise location for some participants.

Access to at-home aerobic exercise equipment such as a treadmill or stationary bike appears to be key to facilitating aerobic exercise during lockdown restrictions. In both groups, walking outside was not a viable option during lockdown restrictions for various reasons, such as uneven terrain, heat, mask wearing, and safety concerns. For many participants, the physical terrain of their local neighborhood was not conducive to walking with a mobility disability. The availability of public spaces such as parks or botanical gardens for aerobic exercise also varied among participants, although most reported closing. In this regard, although our study focused on formalized exercise, these findings have accessibility implications for PA more broadly for people with disabilities desiring to undertake leisure and PA in their homes and communities. Future studies could further examine the role of the environment and neighborhood on PA participation during lockdown restrictions.

Our findings provide additional support for the inclusion of SCT in exercise interventions to enhance program uptake and adherence. SCT posits an interplay between personal and environmental factors and identifies self-efficacy, outcome expectations, goal setting, and facilitators or barriers as core determinants of behavior [32,33]. The STEP for MS exercise program is supplemented with an SCT-based behavioral component that incorporates these core SCT determinants into program tools and strategies. The GEMS-T participants referred to several of these tools and strategies as facilitators of exercise during the lockdown restrictions, including the one-to-one coaching sessions for feedback and social accountability, exercise adherence logs and pedometers for accountability and monitoring progress, informational newsletters discussing SCT determinants, and other individualized strategies to enhance exercise adherence and compliance (eg, listening to music). The participants also discussed factors that were self-rewarding, such as decreased fatigue and stress, greater stamina, and weight loss, as facilitators of continued exercise during the COVID-19 lockdown restrictions.

In line with the previous literature examining SCT and PA [34], our findings suggest that in-person social support may be a latent construct that strongly supports exercise behavior in people with MS. Within the SCT framework, social support facilitates self-efficacy [33], which in turn is a key determinant of behavior change [32,35]. In addition to lacking access to a treadmill, the GEMS-S participants frequently attributed the loss of in-person support received from both coaches and peers as a barrier to continuing the exercise program at home. Although the trial was designed as an individual exercise intervention with identical exercise and coaching content in both study arms, our findings are suggestive of experiential differences between these 2 groups of participants, particularly in perceptions of social support. Although both groups of participants desired social support, the impact of not having it differed qualitatively between the groups, with only the GEMS-S participants naming the loss of support as an impediment to exercise. The outcome data from the trial will provide more conclusive evidence on whether in-person or web-based coaching is associated with differences in exercise outcomes and what role the choice of exercise location plays. Regardless of the impact on the motivation to exercise, several participants requested a way to connect with their peers on the web for social support.

### Recommendations for Research and Practice

These findings are important for funding agencies, exercise scientists, kinesiologists, and rehabilitation professionals who are extending the pandemic-driven growth of telerehab programs for people with disabilities and point to several recommendations. First, an assessment of people’s home and community environment before exercise initiation may be essential for program adherence and success. Discussion with a behavioral coach about safe locations to perform both aerobic and resistance exercises is a core element of the GEMS-T protocol. For exercise interventions without individualized coaching, providing tips and strategies for overcoming commonly encountered barriers to exercising at home or in the community would be helpful.

Second, rehabilitation, exercise science, and kinesiology providers serving people with disabilities and other immunocompromised populations should address COVID-19-specific concerns about exercising. The science of COVID-19 transmission has advanced since these interviews were completed, and except for the most crowded outdoor situations, exercising outside is a low-risk activity for COVID-19 transmission [36,37]. However, exercise guidelines should be updated to include guidance for vulnerable populations on safely exercising indoors, which, as this study shows, is a preference for many participants.

Finally, telerehab programs may wish to consider creating web-based support communities where participants can interact to increase exercise motivation and accountability and decrease isolation and loneliness.

### Limitations

Initially, the GEMS-S participants were informed that they would receive no remote coaching to maintain the integrity of the trial and intervention, and they were encouraged to exercise at home using the provided exercise equipment and study materials. After 3 weeks, the trial co-investigators determined that research participants would be best served by altering the intervention to provide the GEMS-S participants with coaching support remotely to more accurately reflect the original GEMS-S condition. It is impossible to know whether this 3-week gap contributed to the differences in findings between the GEMS-S and GEMS-T groups.

This study used a small convenience sample of 8 participants from the southeast United States and is not representative of the experience of all people in the STEP for MS trial. Therefore, readers should be cautious in extrapolating the findings to all people with MS. The small sample may also obscure other factors that contribute to the differences in experience between the 2 arms of the study, including sociodemographics and geographic and community location. In addition, our sample consisted of only women. Although MS does affect women more than men, gender may influence exercise behaviors during lockdown restrictions.

Additional data may have strengthened our analysis, including data on depression levels, comorbidities, and whether these factors accounted for any exercise differences between the groups. We also did not capture data on the levels of exercise outside the scope of the intervention protocol, although some participants referred to exercising beyond the requirements of the protocol.

The study design randomized participants to 2 groups: either automatic assignment to GEMS-S or GEMS-T or to a group that could choose the study arm. This interview study was unable to detect whether choice had any impact on the experience of the participants during lockdown restrictions. However, the GEMS-S and GEMS-T participants reflected both choice and assigned conditions (Table 1), and the condition did not appear to have had an impact on the exercise experience during lockdown. The STEP for MS trial data analysis will provide insights into the role of choice in exercise outcomes.

### Conclusions

Our findings suggest that lockdown restrictions impacted exercise among people with MS enrolled in a large trial depending on the exercise condition, namely facility based or home based. Although we still do not know the outcome of the larger trial, these findings suggest that the telerehab delivery mode was beneficial to people with MS during lockdown restrictions who continued to exercise uninterrupted. Moreover, people in the GEMS-T study arm reported that the exercise intervention benefited them both mentally and physically. We also learned that social support comes in different forms (coaching vs peers), and this should be explored further and perhaps incorporated into future exercise options. As the United States moves to a new pandemic phase in which federal guidelines endorse shorter isolation time frames and less rigorous masking and social distancing [38], people with MS may still fear resuming prepandemic activities and remain isolated. The boom in telerehab platforms for the remote delivery of exercise interventions has the potential to overcome the barriers to traditional, facility-based exercise options if these platforms incorporate best practice recommendations. Considering a future in which COVID-19 is endemic, rehabilitation professionals should incorporate guidelines for people with suppressed immune systems into exercise protocols to address pandemic-related safety concerns.

## Appendix

Multimedia Appendix 1 Interview guide.

## Abbreviations

GEMS: Guidelines for Exercise in People With Multiple Sclerosis
GEMS-S: Guidelines for Exercise in People With Multiple Sclerosis-Supervised
GEMS-T: Guidelines for Exercise in People With Multiple Sclerosis-Telerehabilitation
MS: multiple sclerosis
PA: physical activity
QOL: quality of life
RTA: reflexive thematic analysis
SCT: social cognitive theory
STEP for MS: Supervised Versus Telerehabilitation Exercise Program for Multiple Sclerosis
telerehab: telerehabilitation
UAB: University of Alabama at Birmingham
