# Supplementary material for: The Impact of COVID-19 Lockdown Restrictions on Exercise Behavior Among People With Multiple Sclerosis Enrolled in an Exercise Trial: Qualitative Interview Study
Source: JMIR Rehabil Assist Technol. 2022 Nov 22;9(4):e42157. doi: 10.2196/42157 (PMC9683526; doi:10.2196/42157)
Supplement: Multimedia Appendix 1 [file rehab_v9i4e42157_app1.docx]

**Appendix**

**Interview guide**

**Grand Tour Question**

1. Please tell me about yourself

Probes:

MS journey

General life – work, family, hobbies

What is your typical week?

**Exercise**

1. Please describe what if any exercise you did prior to STEP for MS

1. [*Clarify participation status when COVID-19 restrictions hit e.g., completed exercise, almost completed, halfway through*]

How did the COVID-19 pandemic initially impact your exercise?

Probes:

1. How much you exercise per week
2. Where you exercise
3. Perceptions on exercise e.g., fear, anxiety, stress, resilience, confidence, motivation
4. Challenges and barriers encountered
5. Any benefits encountered?

1. As the pandemic has continued, how has COVID-19 the impacted your exercise?

Probes:

1. Has it changed in any way?
2. How much you exercise per week
3. Where you exercise
4. Perceptions on exercise e.g., fear, anxiety, stress, resilience, confidence, motivation
5. Challenges and barriers encountered
6. Any benefits encountered?

1. What has helped you exercise during the pandemic?

1. What has been the biggest hindrances to exercise during the pandemic?

1. What additional supports/ tools/ resources would have been helpful during the pandemic?

1. To what extent has the pandemic changed your thoughts about exercise?

Probes:

1. Preference about place of exercise
2. Opinion about benefits of exercise
3. Fears about exercising in the community
4. Preference about level of supervision/coaching
5. Barriers and facilitators to exercise

1. What concerns do you have about continuing in the STEP for MS trial as the pandemic continues?

1. How has COVID-19 changed your thoughts on participating in future exercise or rehabilitation studies?

       Probes:

1. Depends on location (home/facility)
2. Depends on technical resources needed or provided
3. Depends on level of coaching provided

**Wrap up**

1. Is there anything you think I have missed that you would like to discuss, or do you have any questions for me?
